# Nebulous without *white*: annotated long-read genome assembly and CRISPR/Cas9 genome engineering in *Drosophila nebulosa*

**DOI:** 10.1093/g3journal/jkac231

**Published:** 2022-09-05

**Authors:** Christopher J Sottolano, Nicole T Revaitis, Anthony J Geneva, Nir Yakoby

**Affiliations:** Center for Computational and Integrative Biology, Rutgers, The State University of New Jersey, Camden, NJ 08103, USA; Center for Computational and Integrative Biology, Rutgers, The State University of New Jersey, Camden, NJ 08103, USA; Center for Computational and Integrative Biology, Rutgers, The State University of New Jersey, Camden, NJ 08103, USA; Department of Biology, Rutgers, The State University of New Jersey, Camden, NJ 08103, USA; Center for Computational and Integrative Biology, Rutgers, The State University of New Jersey, Camden, NJ 08103, USA; Department of Biology, Rutgers, The State University of New Jersey, Camden, NJ 08103, USA

**Keywords:** genome sequencing, CRISPR/Cas9, genome editing, PacBio

## Abstract

The diversity among *Drosophila* species presents an opportunity to study the molecular mechanisms underlying the evolution of biological phenomena. A challenge to investigating these species is that, unlike the plethora of molecular and genetics tools available for *D. melanogaster* research, many other species do not have sequenced genomes; a requirement for employing these tools. Selecting transgenic flies through *white* (*w*) complementation has been commonly practiced in numerous *Drosophila* species. While tolerated, the disruption of *w* is associated with impaired vision, among other effects in *D. melanogaster*. The *D. nebulosa* fly has a unique mating behavior which requires vision, and is thus unable to successfully mate in dark conditions. Here, we hypothesized that the disruption of *w* will impede mating success. As a first step, using PacBio long-read sequencing, we assembled a high-quality annotated genome of *D. nebulosa*. Using these data, we employed CRISPR/Cas9 to successfully disrupt the *w* gene. As expected, *D. nebulosa* males null for *w* did not court females, unlike several other mutant strains of *Drosophila* species whose *w* gene has been disrupted. In the absence of mating, no females became homozygous null for *w*. We conclude that gene disruption via CRISPR/Cas9 genome engineering is a successful tool in *D. nebulosa*, and that the *w* gene is necessary for mating. Thus, an alternative selectable marker unrelated to vision is desirable.

Significance statement

Morphological and patterning diversities are common in nature. However, the mechanisms underlying these evolutionary differences have been studied only in a limited number of animals. High-throughput tools have been created to study development and evolution, yet the absence of high-quality genome sequences for many organisms of interest has been an obstacle to the exploration of mechanisms controlling diversity in nature. Here, we generated the first high-quality genome sequence of *Drosophila nebulosa* and employed genome engineering to test whether vision is necessary for mating.

## Introduction

The fruit fly *Drosophila melanogaster* has been a leading model system to study genetics and developmental biology. The large mutational screens performed in the 1980s (Lewis *et al.* 1980; Nusslein-Volhard and Wieschaus 1980; Spencer *et al.* 1982; Schupbach and Wieschaus 1986; St Johnston 2002), together with the plethora of effective genetic tools (Duffy 2002; del Valle Rodriguez *et al.* 2011), revealed the functions of many genes, gene regulatory networks, as well as demonstrated how organisms that are phenotypically unrelated share a large proportion of their genes and fundamental molecular and cellular functions (Holley *et al.* 1995; Pearse and Tabin 1998). The introduction of the CRISPR/Cas9 system for targeted and precise genome editing of *D. melanogaster* (Gratz *et al.* 2013) provided an efficient new system to directly manipulate genes and study their influence on organismal phenotypes without the tedious mutation and screening cycles. At the same time, there are thousands of other *Drosophila* species with interesting differences in behavior, chromosomal arrangement, gene expression, pigmentation, diverse cell signaling, and fascinating morphologies (Spieth 1952; Nakamura *et al.* 2007; Kagesawa *et al.* 2008; Schaeffer *et al.* 2008; Markow *et al.* 2009; Niepielko *et al.* 2011; Werner *et al.* 2018, 2020).

Studying the evolution of species at the molecular level is restricted by the availability of their high-quality genome assemblies. A few sequenced *Drosophila* species have limited tools for genetic analyses (i.e. Holtzman *et al.* 2010; Werner *et al.* 2010; del Valle Rodriguez *et al.* 2011; Niepielko and Yakoby 2014; Stern *et al.* 2017), which presents an impediment to understanding the evolutionary mechanisms responsible for common and unique organismal traits. The improvement of sequencing technologies, including long-read sequencing via Pacific Biosciences (PacBio) and Oxford Nanopore, have allowed for massive efforts to sequence the genomes of many different species, including *Drosophila* (Kim *et al.* 2020), as well as updating and improving the contiguity and completeness of existing assemblies (Paris *et al.* 2020). These advances are monumental in furthering the development of biological systems in other *Drosophila* species, which are the stepping stone to study mechanisms of evolutionary diversity.

Since the discovery of a white-eyed fruit fly in 1910 by Thomas Hunt Morgan (Morgan 1910), the *w* gene has been extensively studied in *D. melanogaster*. The gene encodes an ATP-binding cassette transporter, which forms heterodimers with the Scarlet or Brown proteins to deliver pigment precursors into pigment cells, consequently making red eyes in wild-type flies (Sullivan and Sullivan 1975; Sullivan *et al.* 1979). Mutation of this gene may result in the alteration of the protein structure and lead to loss of function, resulting in white-eyed flies (Mackenzie *et al.* 1999). Due to the simplicity of identification, eye-color of *w*-disrupted flies is frequently used as a selectable marker for transgenic flies. However, deleterious effects due to the loss of *w* have been uncovered in the past decades. Several studies have documented alterations in courting, copulation success, exploratory behavior, visual acuity, learning and memory of thermal stress, and sexual preference in *D. melanogaster* overexpressing or deficient of the White protein (Anaka *et al.* 2008; Sitaraman *et al.* 2008; Krstic *et al.* 2013; Ferreiro *et al.* 2017; Xiao *et al.* 2017).

Behavioral changes, such as these have been shown to be the result of altered levels of specific neurotransmitters, such as serotonin and dopamine (Becnel *et al.* 2011; Ries *et al.* 2017), whose precursors are transmitted by the White protein (Krstic *et al.* 2013; Xiao *et al.* 2017). Other studies have reported that *w*-disrupted *D. melanogaster* lack optical insulation provided by eye pigment and thus show impaired visual acuity (Kalmus 1943), increased light sensitivity (Wu and Wong 1977), deficient contrast perception (Wehner *et al.* 1969), atypical phototactic response and electroretinogram (Pak *et al.* 1969; Stark and Wasserman 1972), as well as progressive retinal degeneration (Ambegaokar and Jackson 2010). At the same time, there are numerous white-eyed lines of *Drosophila* species that are viable and used in genetic studies (Holtzman *et al.* 2010).

The fly species *D. nebulosa* belongs to the *willistoni* group (Pavan 1946; Schaeffer *et al.* 2008). This fly has been an attractive system to study the evolution of mating behavior (Spieth 1952; Gleason *et al.* 2012), cell signaling, gene patterning, and eggshell morphology (Niepielko *et al.* 2011, 2014; Niepielko and Yakoby 2014). In *D. nebulosa*, male fruit flies court by producing an anal droplet as a nuptial gift to the female, and subsequently fanning it in their direction with one wing (Spieth 1952; Steele 1986). Unlike other *Drosophila* species, *D. nebulosa* requires vision to locate females in order to initiate mating (Spieth 1952; Keesey *et al.* 2019). In addition, *D. nebulosa* males placed in constant darkness were incapable of inseminating any females (Gleason *et al.* 2012). Since *w* participates in the vision process in flies, we hypothesize that decreased visual acuity, caused by the disruption of *w* (Xiao *et al.* 2017) will impair *D. nebulosa* males’ ability to recognize potential mates, rendering them unable to reproduce.

As a first step to testing the visual requirements underlying mating success in *D. nebulosa* on a molecular level, we generated a high-quality long-read genome assembly using PacBio sequencing. We produced de novo preliminary assemblies using 4 different programs, corrected with short-read Illumina sequencing data, and subsequently merged them into a single hybrid assembly. Gene annotation was then carried out on the assembly, and chromosome synteny was mapped, using *D. willistoni* as a reference. Based on the genomic information, we utilized CRISPR/Cas9 to successfully target the *w* gene in *D. nebulosa*. Independent white-eyed transgenic flies were then validated to ascertain that *w* was disrupted via nonhomologous end-joining. While responding to phototaxis, we observed that, unlike the many other *Drosophila* stocks with white eyes, *w*-disrupted *D. nebulosa* males did not attempt to mate with females.

## Materials and methods

### Fly stocks

The wild-type *D. nebulosa* stock #14030-0761.06 (Isoteca-48) was obtained from the National *Drosophila* Stock Center at Cornell University. Oregon R (OreR) Bloomington #25211 was used as a wild-type *D. melanogaster* stock. Stocks were kept at room temperature (∼22–24°C) and standard cornmeal fly food.

### Genomic DNA extraction and sequencing

Genomic DNA (gDNA) was extracted using a modified protocol provided by the VDRC Stock Center (https://stockcenter.vdrc.at/images/downloads/GoodQualityGenomicDNA.pdf). Male and female *D. nebulosa* heads were used for gDNA extractions bound for PacBio sequencing, and whole male flies were used for Illumina sequencing. In short, tissues (heads or whole flies) were homogenized and incubated in a 0.1-M Tris–HCl/0.1 M EDTA/1% SDS solution and 10 µg RNase A at 70°C for 30 min. Then, 8 M KAc was added, and heads were incubated for another 20 min. Supernatant was phenol–chloroform extracted twice, pelleted using isopropanol and ethanol, in series, and then eluted in nuclease-free water. The gDNA was evaluated for quality on a 0.9% agarose gel (run for 45 min at 100 V) and quantified using a NanoDrop 2000 spectrophotometer (Thermo Scientific).

Pacific Bioscience single molecule sequencing (PacBio) was carried out at the Waksman Genomics Core Facility, Rutgers, The State University of New Jersey. The DNA was quantitated using the Qubit 2.0 instrument and Fragment Analyzer with a DNF-467 Genomic DNA 50 kb Analysis Kit according to the manufacturer’s instructions (Agilent Technologies). Samples were purified using AMPure XP Clean beads (Agencourt Bioscience Corp., Austin, TX, USA). Sequencing libraries were constructed following the manufacturer’s protocol and sequenced on single-molecule real-time (SMRT) cells within a PacBio Sequel System, using version 3.0 chemistry and 10-h runs. Raw reads were generated by combining outputs of 4 sequencing runs, which were carried out using this method. Reads shorter than 3 kb were filtered out using cutadapt v1.8 (Martin 2011).

Genomic DNA was prepared for short-read sequencing, using the NEBNext Ultra II FS DNA Library Prep Kit for Illumina (New England Biolabs). The samples were sequenced as paired-end 2 × 100 nt reads on the Illumina MiSeq platform at the Lewis-Sigler Genomics Core Facility, Princeton University. The FASTQ file was generated, using Illumina MiSeq Control Software under default settings. Only pass-filter reads were used for further analysis.

### Preliminary genome assembly

To account for different biases in genome assemblers, preliminary de novo assemblies were constructed using 4 different programs. All assembly file names, with brief descriptions, can be found in Supplementary Table 1. First, raw reads were corrected, trimmed, and assembled with Canu v2.1 (Koren *et al.* 2017, 2018; Nurk *et al.* 2020) default parameters (except *-genomeSize = 222m*), to generate *neb_c*. For the second preliminary assembly, raw PacBio reads were initially self-mapped (setting *-x ava-pb*), using minimap2 to detect overlaps (Marçais *et al.* 2018), and then concatenated into unitigs using miniasm (Li 2016) to generate *neb_m1*. Raw reads were then mapped back against *neb_mi* using minimap2, generating unpolished and uncorrected contig sequences, *neb_m2*. Racon (Vaser *et al.* 2017) was then used to generate genome consensus of the uncorrected assembly (with inputs *<sequences>=Raw pacbio reads, <overlaps>=neb_m2, <target sequences>=neb_m1*), generating *neb_r1*. Raw reads were additionally mapped against *neb_r1* using minimap2, creating *neb_m3*. Once again, racon was run (with inputs *<sequences>=Raw pacbio reads, <overlaps>=neb_m3, <target sequences>=neb_r1*) to generate the final corrected a genome consensus, *neb_m*. The third assembly used Flye (Kolmogorov *et al.* 2019) to assemble and polish raw reads on default settings (except *–pacbio-raw*), generating *neb_f*. Finally, raw reads were also assembled using wtdbg2 (Ruan and Li 2020) with default settings (except *-x sq, -g 222m*) to create *neb_w1*. The final consensus, *neb_w*, was then generated using wtpoa-cns (Ruan and Li 2020) with default settings.

### Genome polishing

Preliminary assemblies were then polished using short-read Illumina sequencing data from *D. nebulosa*. Short-read data were aligned to each individual preliminary assembly, using BWA (Li and Durbin 2009). The resulting SAM file was converted to the BAM format, sorted, and indexed using Samtools (Li *et al.* 2009) with default settings. This resulting file, as well as its respective preliminary assembly, were then input into Pilon (Walker *et al.* 2014) for polishing. Parameters were set as diploid, but otherwise kept default. As a note, a “*p*” was appended to the end of Pilon-corrected preliminary and composite *D. neb* assemblies (e.g. Pilon-corrected *neb_w* was named *neb_wp*) (Supplementary Table 1).

### Assembly merging

**Fig. 1.**
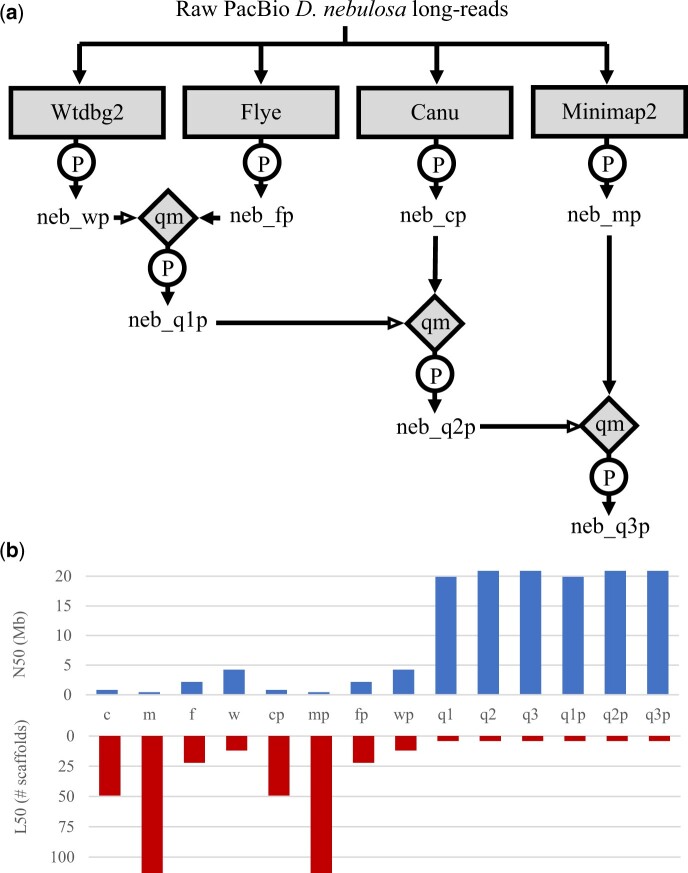
Composite assemblies showing improved contiguity. a) Flowchart of the *D. nebulosa* genome assembly. To account for different biases among genome assemblers, preliminary de novo assemblies were generated using four different programs (rectangles). Preliminary assemblies were polished with Pilon, using short-read Illumina sequencing data from *D. nebulosa* (circles), and then merged into composite assemblies using quickmerge (diamonds). This was done by assigning the query assembly as the most contiguous, and the reference assembly as the second most contiguous. Hollow arrow heads indicate the assembly used as the query into which the subject assembly was merged. b) Comparison of assembly contiguity. The N50 (top) is the length of the shortest contig at 50% of the total genome length, while the L50 (bottom) is the number of contigs whose lengths sum up to half the genome size. The x-axis shows different versions of the *D. nebulosa* assembly, named according to the preliminary or composite assemblies (see Figure 5): c = Canu, m = Minimap2, f = Flye, and w = Wtdbg2, q = Quickmerge (composite). A ‘p’ was appended to the end of Pilon-corrected preliminary and composite *D. nebulosa* assemblies.

Preliminary assemblies were then combined into hybrid assemblies, using quickmerge (Chakraborty *et al.* 2016) and MUMmer (Marçais *et al.* 2018). This was done by assigning the query assembly as the most contiguous, and the reference assembly as the second most contiguous. For example, out of the 4 preliminary assemblies, *neb_wp* was the most contiguous, and *neb_fp* was the most complete. As such, they were selected as the query and reference when generating the initial composite assembly (*neb_q1p*), respectively. The specific order of merging is detailed in Fig. 1a. Minimum seed contig length to be merged (length cutoff) was set to 500. Composite assemblies were again polished using short-read Illumina data in Pilon between each merge, as described in the previous section.

### Contiguity and quality

Assembly statistics (Supplementary Table 1) were calculated, using the abyss-fac function from ABySS v2.1.5 (Jackman *et al.* 2017) and stats function from BBMap v38.87 (Bushnell 2014). Assembly completeness (Supplementary Table 2) was evaluated using BUSCO v5.1.2 (Simao *et al.* 2015) to compare gene content in preliminary and composite assemblies to the diptera_odb10 lineage dataset (specifically, *-l diptera_odb10* and *-m genome*). The diptera_odb10 lineage dataset set contains 3,285 orthologs found to be present and single copy across 56 dipteran genome assemblies performed to date. The presence of orthologous genes in their complete form, and without duplication, allows us to assess how complete our assemblies are with respect to gene content. Contiguity and completeness of reference *Drosophila* assemblies were assessed using the same methods.

### Gene annotation

We used the Maker v2.31.11 pipeline (Cantarel *et al.* 2008; Campbell *et al.* 2014) to annotate the polished final composite assembly (*dneb_q3p*) (Fig. 4a). For the initial run, parameters we edited in the *maker_opts.ctl* data file as described below, otherwise set to 0, or left blank. The polished composite *D. nebulosa* assembly was used for annotation in FASTA format (*<genome>=neb_q3p*, *<organism_type>=eukaryotic*). Proteomes obtained from UniProt of species *D. melanogaster* (GenBank reference: GCA_000001215.4), *D. pseudoobscura* (GCF_009870125.1), and *D. willistoni* (GCA_000005925.1) were provided as protein homology evidence in FASTA format (*<protein>=mel_prot*, *pse_prot*, *wil_prot*). The Repbase repeat library from *D. willistoni* (Jurka 1998, 2000) was used as a model organism for soft repeat masking (*<model_org>=Drosophila_willistoni*, *softmask = 1*). Gene prediction was inferred only using protein homology (*<protein2genome = 1*). Lastly MAKER behavior settings were set (with inputs *<alt_peptide>=C*, *<cpus>=1*, *<max_dna_len>=200,000*, *<min_contig>=2,000*, *<pred_flank>=200*, *<AED_threshold>=1*, *<split_hit>=10,000*, *<tries>=5*).

We ran MAKER in a Singularity Biocontainer distributed by Bioconda (https://bioconda.github.io/). Repeat masking was performed using RepeatMasker v4.1.1 (Smit *et al.* 2013–2015). Initially, MAKER was used for ab initio gene prediction, as well as aligning protein evidence to *neb_q3p* (with MAKER flags *-fix_nucleotides* and -*nodatastore* and Singularity flags *–no-home* and *–cleanenv*). MAKER then used 2 gene annotation programs to integrate evidence and produce gene models: SNAP (Korf 2004), and Augustus v3.4.0 (Stanke *et al.* 2008). Both were then trained using the resultant predictions, with SNAP specifying an AED and amino acid length of 0.25 and 50 or greater, respectively (*maker2zff -x 0.25 -l 50*). BUSCO was used to train Augustus (*with inputs -l diptera_odb10 -m genome -c 30 –augustus –augustus_species fly –long –augustus_parameters=*’*—progress=true*’) on mRNA annotated regions flanked on either side by an additional 1,000 bp.

MAKER was then rerun to improve on the existing gene models by replacing previous evidence with the newly generated SNAP and Augustus models (retraining parameters). Additionally, tRNAscan-SE (Chan and Lowe 2019) was enabled for the detection and annotation of tRNAs. The maker_opts.ctl file was altered in the following ways: *<protein_gff>=rnd1.protein2genome.gff*, *<rm_gff>=rnd1.repeats.gff*, *<snaphmm>=dneb1.l50.aed24.hmm*, *<est2genome>=0*, *<protein2genome>=0*, *<trna>=1*. MAKER was run a total of 4 times, each time replacing the repeat GFF file and SNAP HMM with that of the previous run. After each iteration, the models were evaluated for BUSCO completeness, number of gene models, and AED distribution. BUSCO was run using the transcript FASTA and Augustus retraining parameters of each respective MAKER iteration (*with inputs -l diptera_odb10 -m transcriptome -c 8 –augustus_species Dnebulosa –augustus_parameters=*’*—progress=true*’) (Supplementary Table 3). Specifically, the AED distribution (Supplementary Table 4 and Fig. 4e) was calculated using AED_cdf_generator.pl (https://github.com/mscampbell/Genome_annotation/blob/master/AED_cdf_generator.pl), by using the master GFF file as the input, and specifying the bin size (*-b 0.025*).

Gene model IDs were renamed and mapped using MAKER’s *maker_map_ids*, *map_gff_ids*, and *map_fasta_ids* functions, following a protocol described in the section *Renaming Genes for GenBank Submission* in Campbell *et al.* (2014). The proteome of *D. melanogaster* was used as a BLAST reference to obtain names for *D. nebulosa* orthologs, using protocol described in section *Assigning putative gene function* (Campbell *et al.* 2014). Gene names were mapped to model IDs via Annie (Tate 2014), using the aforementioned *D. melanogaster* proteome and BLAST results (Supplementary Table 4). Finally, Genome Annotation Generator (GAG) (Geib *et al*. 2018) was used to rename gene models, as well as to pull annotation statistics. *Drosophila nebulosa* gene models were protein-aligned, via *blastp*, against the *D. willistoni* proteome as an additional measure of ortholog homology.

### Phylogenomic tree building

We used the BUSCO_phylogenomics pipeline (McGowan *et al.* 2020; McGowan and Fitzpatrick 2020) to assess the phylogenomic position of our *D. nebulosa* assembly with respect to related species with genome assemblies available. This pipeline involves first running BUSCO on any genomes to be included in the phylogeny to find orthologous genes for phylogenomic tree building. Using the same BUSCO settings described above, we analyzed 26 genome assemblies from 23 species (Supplementary Table 8) (Clark *et al.* 2007; Zimin *et al.* 2008; Hoskins *et al.* 2015; Chakraborty *et al.* 2017; Zhang, Yu, *et al.* 2018; Liao *et al.* 2019; Paris *et al.* 2020; Pinharanda *et al.* 2020; Reilly *et al.* 2020). Assemblies from the *D. willistoni* and *D. saltans* subgroups were also included to ensure adequate phylogenomic comparison within and to adjacent monophyletic branches (Kim *et al.* 2020).

Results for each BUSCO run were then used by the BUSCO_phylogenomics pipeline to create trimmed alignments for each gene that was present and single copy in every queried genome. Alignments were performed using MUSCLE v 3.8.31 (Edgar 2004a, 2004b) and trimmed using TrimAl v 1.4 (Capella-Gutierrez *et al.* 2009). These alignments were then used to infer phylogenies by either concatenation and phylogenetic analysis in IQ-Tree v1.2.12 (Nguyen *et al.* 2015) (supermatrix method) or by inferring individual gene trees with IQ-TREE and performing species tree inference using ASTRAL v. 5.7.7 (Zhang, Rabiee, *et al.* 2018) (supertree method). We ran BUSCO_phylogenomics with default settings, except that IQ-TREE (Nguyen *et al.* 2015) was run using the *-safe* flag. All trees were rooted, using *Scaptodrosophila lebanonensis* and visualized using iTOL (Letunic and Bork 2021).

### Chromosome synteny

We next placed *D. nebulosa* scaffolds to predicted chromosomes by comparing conserved loci to a reference genome. *D. willistoni* was chosen as a reference due to the genome's similar chromosomal arrangement to *D. nebulosa*. As a note, the *D. willistoni* caf1 (GCA_000005925.1) and *D. willistoni* 17 (GCA_018903445.1) assemblies will hereby be referenced as *wil_caf1*, and *wil_17*, respectively. First, we identified chromosomal locations of *wil_caf1* assembly scaffolds, using data from previous studies (Supplementary Table 9) (Schaeffer *et al.* 2008; Garcia *et al.* 2015). In an attempt to use a more contiguous reference, we used Satsuma2 (https://github.com/bioinfologics/satsuma2) (Grabherr *et al.* 2010) to find syntenic scaffolds between the *wil_caf1* and *wil_17* assemblies (Supplementary Table 10). This was done specifically by comparing only chromosome-annotated *wil_caf1* scaffolds to the 20 largest *wil_17* scaffolds. From this output, *wil_17* scaffolds syntenic to those of *wil_caf1* were selected, renamed according to chromosome (Supplementary Table 11), and then compared with 11 largest *D. nebulosa* scaffolds using Satsuma2 (Supplementary Table 12). As an additional evaluation, annotated *wil_caf1* scaffolds were also compared with the 11 largest *D. nebulosa* scaffolds using Satsuma2 (Supplementary Table 13). All Satsuma2 comparisons were run with default settings. The R circlize package was used to visualize chromosome synteny between *D. nebulosa* and *D. willistoni*, using a representative subset of alignments with identities greater than 0.75. Only *D. willistoni* scaffolds with over 2,000 aligned regions with *D. nebulosa* were visualized (Fig. 5 and Supplementary Fig. 1).

### Transposable element mapping

Transposable element density was mapped to the 11 largest scaffolds to predict centromeric locations. To map transposable elements in the *D. nebulosa* genome, we used the Bedtools v2.50.0 (Quinlan and Hall 2010) makewindows function to divide the largest 11 scaffold lengths in the assembly into 10 kb windows (setting *-w 10000 -s 10000*), generating *neb_win.10k*. We then used LINE, LTR, DNA transposable elements, and helitron MAKER annotated repeats, as well as *neb_win.10k*, as inputs for the Bedtools coverage function, to calculate the number of genes within each 10 kb window. Statistics were visualized for the largest 11 scaffolds using the circlize package (Gu *et al.* 2014) in RStudio.

### CRISPR experimental design

To develop a white-eyed *Cas9*-expressing transgenic *D. nebulosa* fly, we used homology-directed repair to disrupt *w*, while simultaneously inserting *Cas9* under a *nanos* promoter. The CRISPR/Cas9 system works by creating a double-strand break proximal to a specified 20 bp target site adjacent to a protospacer adjacent motif (PAM) sequence, facilitated by a guide plasmid (Gratz *et al.* 2013). Point mutation *w* alleles in *D. melanogaster* from Mackenzie *et al.* (1999) were mapped to exons 3–6 in *D. nebulosa*. The locus was targeted (Fig. 6a) by aligning both sequences in MEGA (Kumar *et al.* 2016) and choosing PAM sites which flank the predicted region. Target cut sites were determined using CRISPR Optimal Target Finder (Gratz *et al.* 2013), and cleavage efficiency was predicted using CRISPR Efficiency Predictor (Housden *et al.* 2015). The formerly mentioned program was used to find target sequences adjacent to PAM sites, and to compare them to a reference genome to look for similar off-target cut sites. Since the *D. nebulosa* genome was not listed on the site, we attempted to account for off-target cut sites using the *D. willistoni* genome. The selected target sequences were then aligned via BLASTn to our *D. nebulosa* genome to look for matches.

To repair the double-strand break(s) by the guide(s), a donor vector was designed featuring the *Cas9* gene under the *nanos* promoter (see *CRISPR constructs* section in *Materials and methods*). The vector insert was flanked by two 1,000 bp, arms which are homologous to the *D. nebulosa w* loci surrounding the target region, as described in Gratz *et al.* (2013). Three separate guide injections (Rainbow Transgenics, CA) (Fig. 6c) were used increase the likelihood of a unique target, as well as to the test the efficiency of 1 vs 2 guides*.* All injections included the *Cas9*-containing donor plasmid (1.12 μg/μl) and Cas9 protein (5 μg/μl) (ThermoFisher #A36498). Injections differed in the combination of guide plasmids, where injection #1 contained the neb_w_guide1 plasmid (2.58 μg/μl), injection #2 contained neb_w_guide2 plasmid (1.33 μg/μl), and injection #3 contained neb_w_guide1 and neb_w_guide2 (1.76 μg/μl) (Fig. 6c). All oligonucleotides are shown in Supplementary Table 14.

### CRISPR constructs

Two different guide oligonucleotides were ligated into individual pU6-BbsI-chiRNA plasmids (Melissa Harrison, Kate O'Connor-Giles, and Jill Wildonger; Addgene plasmid # 45946), as described in Gratz *et al.* (2013). The donor vector (Fig. 6a) was designed using a modified Gratz *et al.* (2013) procedure. Left and right homology arms were amplified from genomic DNA of whole *D. nebulosa* flies. The donor vector backbone and *nos*-*Cas9* locus were amplified from a *pnos-Cas9-nos* plasmid (Addgene plasmid # 62208) (Port *et al.* 2014). A complete list of primers used is listed in Supplementary Table 14 under *Primers used for CRISPR*.

All donor vector fragments were ligated into a circular plasmid via New England Biolabs HiFi Assembly Master Mix Gibson Assembly (E2621). The 4-fragment Gibson assembly used a 1:1 vector: insert ratio, containing 148.85 ng *pnos-Cas9-nos* backbone (28.3 ng/μl), 163.84 ng *nos-Cas9* insert (28.2 ng/μl), 31.68 ng right homology arm insert (57.6 ng/μl), and 32.70 ng (54.5 ng/μl) right homology arm insert, for a total reaction volume of 22.23 μl. All plasmids were cloned in DH10β *E. coli* bacteria, and screened by PCR amplification using T3 and T7 primers. Plasmids were extracted and purified using the ZymoPURE II Plasmid Midiprep Kit (Zymo Research). Guide plasmids were all sequence-validated with T3 primers. The donor vector was sequenced using primers w_insF, w_ins2R, and nebRHAwR (independent reactions). Plasmids were digested with SapI exonuclease (CutSmart R0569S) and validated using restriction fragment mapping.

### Crosses and line characterization

CRISPR injected (G0) male and female flies were separated immediately after eclosion from the pupa and mated to wild-type *D. nebulosa*. Progeny (F1) were then screened for white eyes. Wild-type virgin females *D. nebulosa* were then crossed to white-eyed *D. nebulosa* males and left to self-cross in an attempt to establish a white-eyed stock. Red-eyed *D. nebulosa* males were selected against during this process. To validate the CRISPR locus, genomic DNA was extracted from white-eyed males of each positive line, and compared with wild-type *D. nebulosa*. Primers flanking the target region (w_insF, w_ins2R) were used for DNA amplification (Supplementary Table 14, *Primers used for sequencing*). The PCR products were then sequenced and aligned to the *D. nebulosa w* reference locus using MEGA. Mutations/deletions in the *w* gene as well as the presence/absence of *Cas9* were determined.

### Quantification of courtship and phototaxis assay

Wild-type and *white*-disrupted *D. nebulosa* males were individually paired to virgin a female. Vials were video recorded for a span of 4 h, and the footage was analyzed using BORIS (Friard and Gamba 2016) to annotate instances of courting.

We next tested whether white-eyed *D. nebulosa* could sense and respond to light. Flies were enclosed in a 28-cm plastic cylinder, segmented into thirds (labeled 1–3), and left to adjust to darkness for 30 min. Each phototaxis trial was initialized by placing 19 flies into the end of tube 1, leaving them in darkness for 15 min, and then recording the quantity of flies in each segment. Next, a Leica KL 200 LED cold light source set to 0.5 brightness was shone into the distal end of segment 3 for 15 min. To limit the brightness even further, the light was covered by one layer of a paper towel. The quantities of flies in each segment were again recorded. Four trials were conducted for both wild-type, as well as white-eyed male *D. nebulosa*. The experiment was carried out at room temperature (23°C). The experimental setup is visualized in Fig. 7a. Results were analyzed for significance in the dataset, using 1-way ANOVA, and subsequent Tukey HSD tests for all-verses-all comparison of treatment means were performed.

## Results

### Sequencing and de novo genome assembly

**Fig. 2.**
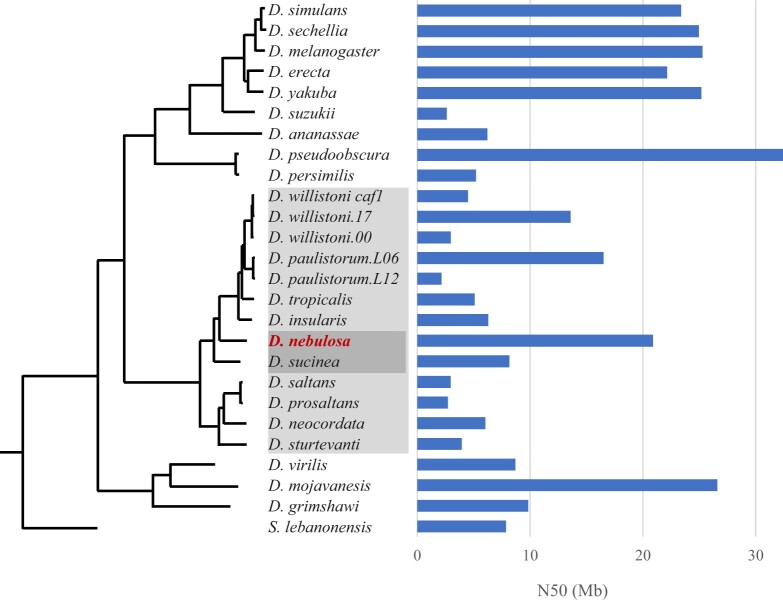
The *D. nebulosa* assembly is highly contiguous compared with other available *Drosophila* assemblies. Shown is a phylogenomic tree of *D. nebulosa* and *Drosophila* assemblies referenced in this work, with associated N50 values. The phylogenomic tree of the *Drosophila* genus shown is based on supermatrix methods. The topology was inferred via concatenation of 3285 Universal Single Copy Orthologs present in all lineages and rooted with *Scaptodrosophila lebanonensis*. Bootstrap values were 100% for all branches. Light and dark shaded species are members of the *willistoni* group and *bocainensis* subgroup, respectively.

**Fig. 3.**
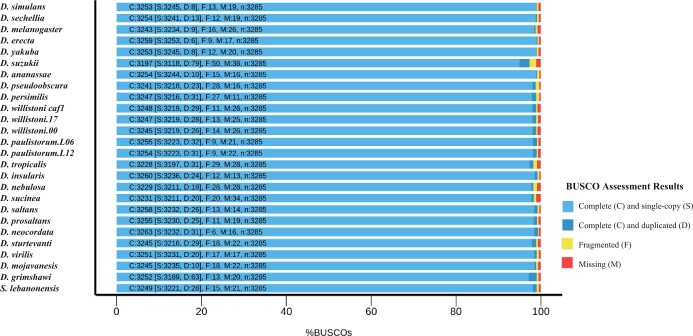
The final *D. nebulosa* assembly shows completeness consistent with other available *Drosophila* assemblies*.* Assembly completeness was evaluated using BUSCO to compare gene content in preliminary and composite assemblies to a dataset set containing 3,285 orthologs found to be present and single copy across 56 dipteran genome assemblies performed to date. The presence of orthologous genes in their compete form, and without duplication, allows us to assess how complete our assemblies are with respect to gene content. The *x*-axis shows the percentage of orthologs (BUSCOs) that are complete and single copy, complete and duplicated, fragmented, and missing in each assembly.

Long-read sequencing generated 13 Gb of sequence from 1,717,740 subreads above 3 kb with a read N50 of 8.4 kb. Short-read Illumina sequencing generated 29,211,787 forward reads, 100 bp in length. To account for different biases in genome assemblers we used 4 separate programs to generate preliminary assemblies, and subsequently merged them in a step-wise fashion (Fig. 1a). Composite assembly *neb_q1p* (merge of *neb_wp* and *neb_fp*) showed considerable improvements in contiguity, as well as completeness similar to *neb_fp*. Subsequent merges with *neb_cp*, and then *neb_mp*, improved the resultant composite assemblies (*neb_q2p* and *neb_q3p*, respectively), though only marginally (Fig. 1b). Contiguity of *neb_q3p* also compares favorably with other available *Drosophila* assemblies in the *willistoni* group (Fig. 2). The final assembly (*neb_q3p*) has a total of 1,600 scaffolds, with an N50 of 20.9 Mb, and a total size of 177 Mb. Statistics for each preliminary and composite assembly are listed in Supplementary Table 1. Analysis of 3,285 universal single copy Dipteran orthologs (BUSCO diptera_odb10 dataset) in *neb_q3p* revealed 98.2% (3,229) to be present and full length (97.7%, 3,211 single-copy; 0.5%, 18 duplicated), 0.9% (28) were present but fragmented, and 0.9% (28) of these genes were missing from our assembly (Fig. 3). Comparative BUSCO scores for our preliminary and final composite assemblies are listed in Supplementary Table 2.

### Genome annotation

**Fig. 4.**
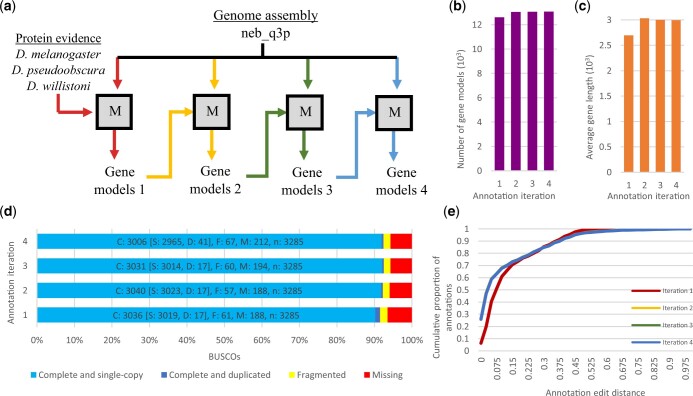
Genome annotation was improved after successive retraining iterations to an extent. a) Flowchart of the *D. nebulosa* genome annotation. The MAKER pipeline (represented by the squares) uses two gene annotation programs (SNAP and Augustus) to integrate evidence and produce gene models. The resultant predictions are then used to train the gene annotation programs, thus iteratively improving the annotation. Initially, MAKER was used to align protein evidence (*D. melanogaster, D. pseudoobscura,* and *D. willistoni* proteomes) to the *D. nebulosa* assembly and produce ab initio gene predictions. Gene models were retrained via the MAKER pipeline a total of four times, each time replacing previous evidence with the newly generated gene models. Red, yellow, green, and blue arrows represent the first, second, third, and forth MAKER iterations. b) Number of gene models, (c) average gene length, (d) annotation completeness (BUSCO score), and (e) annotation edit distance all show improvement by the second MAKER iteration and appear to stabilize by the third. Annotation edit distance (AED), a measurement of how well an annotation agrees with overlapping protein homology evidence (scores 0 and 1, denoting perfect and no agreement to aligned evidence, respectively), shows 97% of the annotation with a score of 0.5 and below. Since the fourth run of the pipeline produced little improvement based on BUSCO scores and AED, the third iteration of gene predictions was chosen as the final annotation. It should be noted in the figure that AED values for iterations two through four overlap.

Gene models were retrained via the MAKER pipeline a total of 4 times (Fig. 4a). Since the fourth run of the pipeline produced little improvement based on number and average length of gene models, BUSCO scores, and annotation edit distance (AED) (Fig. 4, b and e; Supplementary Table 3), the third iteration of gene predictions was chosen as the final annotation and will thus be reported on in this section. Genome annotation through de novo prediction and homology with *D. melanogaster* produced 13,067 gene models (Table 1). Protein BLAST alignments of *D. nebulosa* models with *D. melanogaster* and *D. willistoni* generated 12,548 and 12,578 alignments, respectively, indicating orthology with both species (Supplementary Table 4). BUSCO analysis of the transcriptome revealed 92.5% (3,040) completed (92.0%, 3,023 single-copy; 0.5%, 17 duplicated), 1.7% (57) fragmented, and 5.8% (188) missing orthologs (Fig. 4d) suggesting that our annotation includes the vast majority of genes present in our assembly. AED, a measurement of how well an annotation agrees with overlapping protein homology evidence (scores 0 and 1, denoting perfect and no agreement to aligned evidence, respectively) (Holt and Yandell 2011), shows 97% of the annotation with a score of 0.5 and under (Fig. 4e; Supplementary Table 5).

**Table 1.** *Drosophila nebulosa* genome annotation statistics.

Total sequence length	176.8 Mb
Number of genes	13,067
Number of exons	52,709
Number of introns	39,642
Number of CDS	12,698
Overlapping genes	94
Contained genes	43
Total gene length	39,234,692 bp
Total exon length	21,059,387 bp
Total intron length	18,254,589 bp
Total CDS length	21,016,413 bp
Shortest gene	68 bp
Shortest exon	3 bp
Shortest intron	8 bp
Shortest CDS	84 bp
Longest gene	88,781 bp
Longest exon	13,185 bp
Longest intron	21,319 bp
Longest CDS	68,394 bp
Mean gene length	3,003 bp
Mean exon length	400 bp
Mean intron length	460 bp
Mean CDS length	1,655 bp
% of genome covered by genes	22.2
% of genome covered by CDS	11.9
Mean mRNAs per gene	1
Mean exons per mRNA	4
Mean introns per mRNA	3

### Chromosome synteny and transposable element distribution

**Fig. 5.**
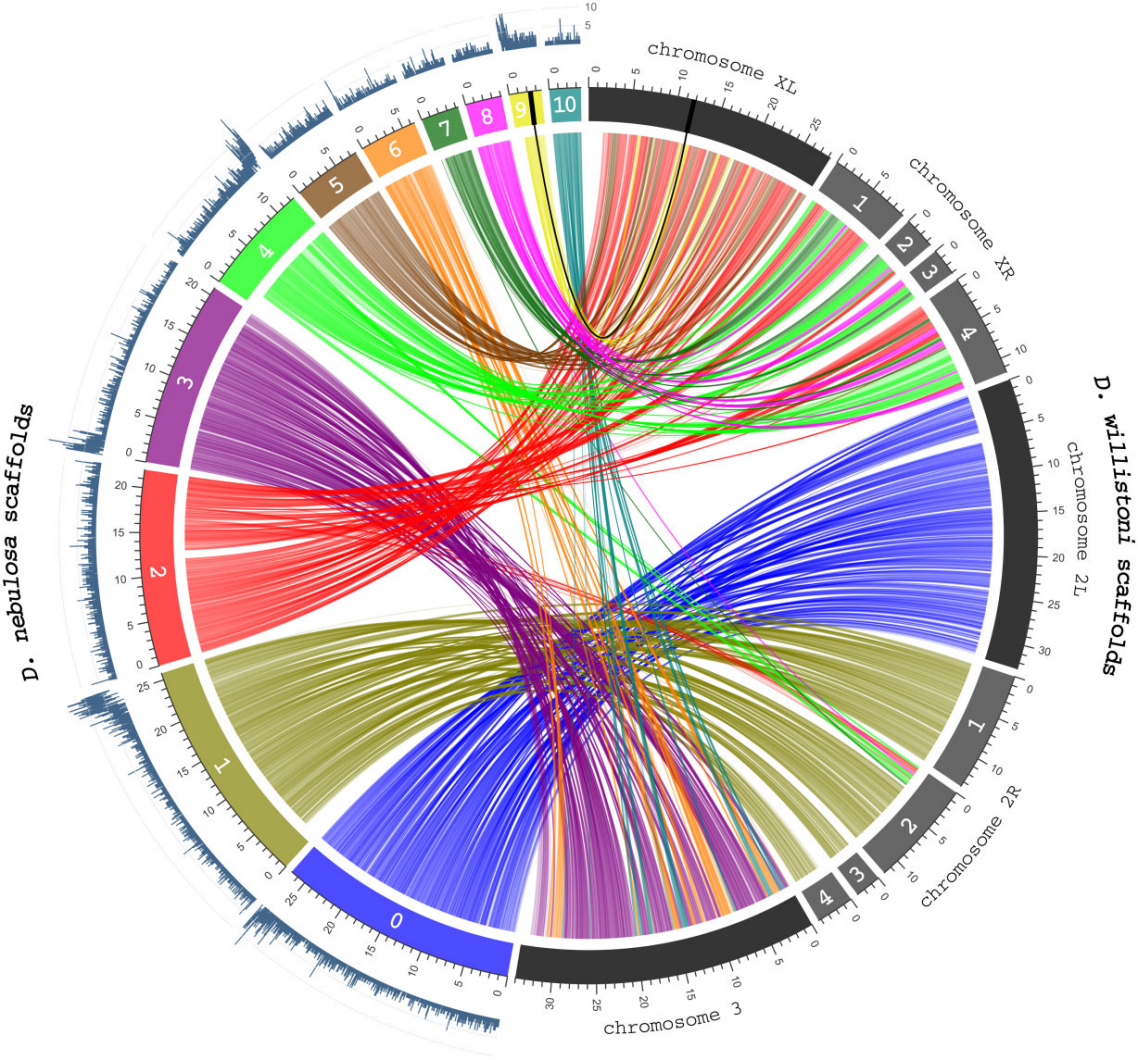
Chromosomal location of *D. nebulosa* scaffolds can be predicted via transposable element density and comparison of conserved loci with *D. willistoni*. The numbers of LINE, LTR, DNA transposable element, and helitron annotated repeats within 10-kb windows (dark blue, outer ring) are mapped to the largest 11 *D. nebulosa* assembly scaffolds (colored segments, middle ring). Scaffolds aligned between *D. nebulosa* and *D. willistoni* (*wil_17*) assemblies were found to be highly syntenic, allowing identification of homology between chromosomes. However, we also observe considerable internal reorganization within chromosomes. Annotated *D. willistoni* scaffolds are grouped by chromosome (indicated by the light and dark gray segments, middle ring) and compared with the 11 largest *D. nebulosa* scaffolds. Syntenic regions between the 2 assemblies are represented by the curved lines and colored according to synteny with an associated *D. nebulosa* scaffold (inner circle). Numbers within *D. willistoni* scaffolds label the order of position on the chromosome arm. Numbers within the *D. nebulosa* scaffolds represent the internal scaffold numbers. Apostrophes denote predicted chromosomal locations. The loci of *w* orthologs and their syntenic connections in *D. nebulosa* and *D. willistoni* assemblies are represented by the bolded lack line. Scales are in Mb.

Scaffolds aligned between *D. nebulosa* and *D. willistoni* (*wil_17*) assemblies were found to be highly syntenic, allowing identification of homology between *D. nebulosa* and *D. willistoni* chromosomes. However, we observe considerable internal reorganization within chromosomes (Fig. 5). *Drosophila nebulosa* scaffolds dneb_sca_0 and dneb_sca_1 are each syntenic with *D. willistoni* scaffolds belonging to a single chromosome arm (Chr2L and Chr2R_1-4, respectively). Others, such as dneb_sca_3, 6, and 10 all appear to constitute the *D. willistoni* chromosome 3 scaffold (Chr3). Consistent with these data, the genes *eyeless* (*ey*) and *cubitus interruptus* (*ci*), which are known to be located on chromosome 3 in *D. nebulosa* (Pita *et al.* 2014), can be found on scaffold *dneb_sca_3* in our assembly. The X chromosome appears to be less contiguous with dneb_sca_2, 5, and 9 syntenic to the *D. willistoni* left arm (ChrXL), and dneb_sca_2, 4, 7, and 8 syntenic to the *D. willistoni* right arm (ChrXR1-4). Of note, dneb_sca_2 appears to span across both arms of *D. willistoni* Chromosome X, with the right arm syntenic to position 22,716–8,714,232, and left to 8,775,313–21,991,806. Syntenic alignment of *D. nebulosa* and *D. willistoni* (*wil_caf1*) assemblies (Supplementary Fig. 1) were largely consistent with the aforementioned data, with the exception of a rearrangement between the right arm of chromosomes X and 2, which differ slightly in position and size.

As a method of determining potential centromere location within the assembly, we mapped all annotated class I and II transposable elements to the largest 11 scaffolds. We see considerable enrichment of transposable elements at the start of dneb_sca_3 and 9, and at the end of dneb_sca_0, 1, and 4 (Fig. 5). A few smaller spikes of transposable elements are interspersed throughout the scaffolds, however, the higher density regions at the scaffold ends indicate these locations as likely centromeres.

### *white* was successfully disrupted in *D. nebulosa*, but not repaired via homology-directed repair

**Fig. 6.**
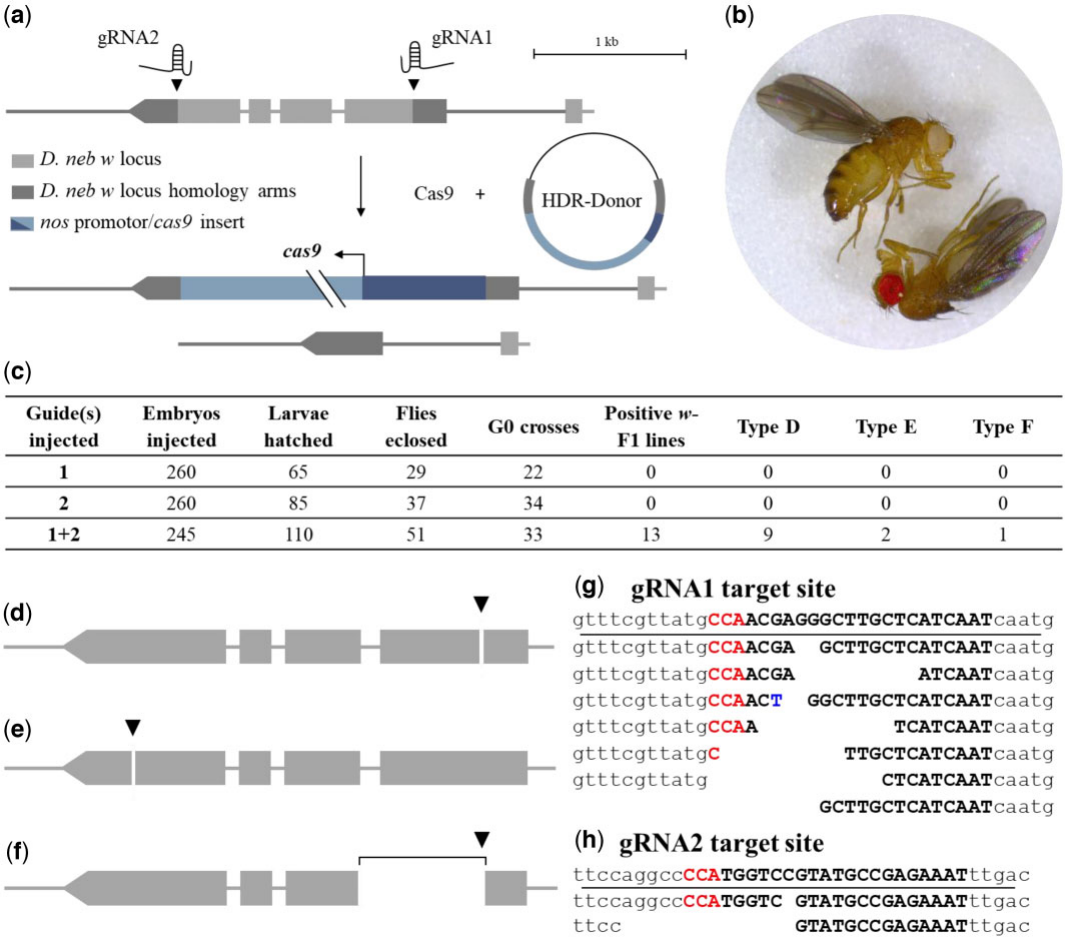
CRISPR/Cas9 was used to disrupt w in the *D. nebulosa* genome via non-homologous end joining. a) Top: Double-strand breaks were made in exon 3 (gRNA1) and 6 (gRNA2), targeting the region in between for removal. Three separate injections of guide(s) 1, 2, and 1+2 were used to facilitate homology-directed repair. Middle: Successful integration of the *Cas9* locus should allow for the endogenous expression of Cas9 protein. Bottom: Alternatively, the w locus may be disrupted without the integration of *nos-Cas9* locus. b) *w*-disrupted male (white eye) and wild-type female (red eye) *D. nebulosa*. c) The table shows results of three separate CRISPR injections using the guides individually and in combination with each other. The number of embryos injected and larvae hatched were obtained from Rainbow. (D - F) CRISPR/Cas9 created deletions in the *D. nebulosa* genome, but did not integrate the *nos-Cas9* locus. The two guides generated double strand breaks that could be generally categorized into three types: (d) a 2–14 bp deletion at the gRNA1 target site, (e) a 1–14 bp deletion at the gRNA2 target site, and (F) a 565 bp deletion downstream of the gRNA1 target site. g, h) Characterization of nucleotide deletions at target sites of gRNA1 and 2, respectively. Reference sequence of the w locus are shown above the line, with the different *w*-disrupted lines shown below. PAM sites are shown in red letters, gRNA target sequences are black bolded letters, and any base mutations are shown in blue letters.

Following genome assembly and gene annotation, we aimed to test the requirement of vision in *D. nebulosa* mating by generating a white-eyed *D. nebulosa*, as a proof of concept. If mutation in *w* can be tolerated, as in other species, we intended to insert the *Cas9* gene into the *w* gene to obtain a stock that can potentially be used for future CRISPR/Cas9 genome engineering. Briefly, the *D. nebulosa w* locus was targeted by a combination of 2 guides, Cas9 protein, and a homology directed repair vector with the *Cas*9 gene (more details can be found in the *Materials and methods*) (Fig. 6a). Since the *w* gene is on the X chromosome, the expectation was to obtain white-eyed male flies hemizygous for disrupted gene. In total, 13 F1 lines of white-eyed *D. nebulosa* males were selected positive for the CRISPR disruption (Fig. 6, b and c). Despite repeated attempts, females remained heterozygous for the *w* null allele, thus the white-eye phenotype was only found in males.

Although the initial intent was to insert the *Cas9* gene into the *w* gene, PCR validation of white-eyed *D. nebulosa* CRISPR target loci revealed that neither insert integration, nor complete deletion occurred in any of the lines (Fig. 6, c–h). Interestingly, lines successful for disruption of *w* were all from embryos injected with both guide plasmids (Fig. 6c). While the 2 guides promoted gene disruption, deletions were only found to be present around one PAM site per line (7 out of the total of 10 achieved from guide 1, Fig. 6g), but never at both. Instead, both guide plasmids created asynchronous 1–14 bp deletions on or upstream of the PAM sites (Fig. 6, d–h). In one case, a 565-bp deletion was characterized adjacent to guide 1 (Fig. 6f).

### *white*-disrupted *D. nebulosa* males respond to light but were not observed courting females

A cross between wild-type females and white-eyed males failed to produce developing embryos. As a result, white-eyed females were never observed in any of the lines. This is supported by the fact that a white-eyed male in this cross was not observed courting the wild-type female even once over a period of 4 h. At the same time, a control cross of a wild-type male and female displayed 13 instances of distinctive courtship. The courtship was observed in varying intervals (42.0 ± 30.6 s) for ∼9.1 min, cumulatively.

**Fig. 7.**
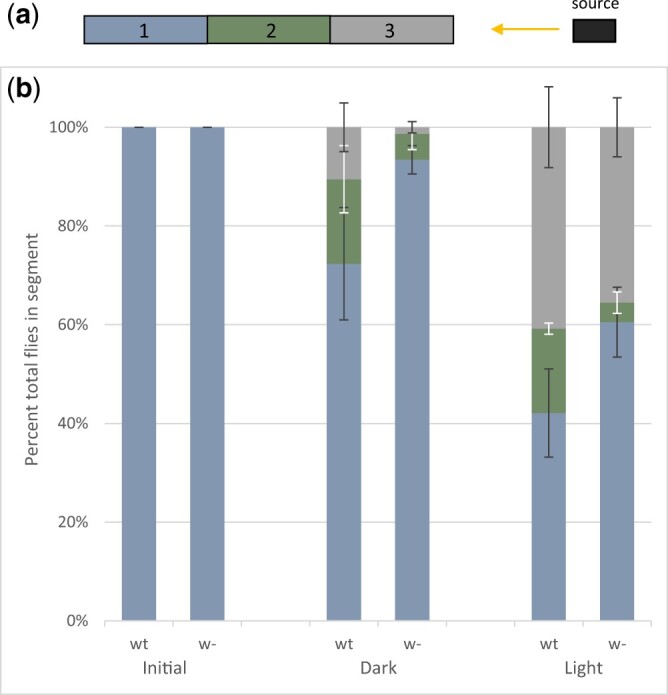
Phototactic response of *D. nebulosa* wild-type and *white*-disrupted flies. a) The experimental setup is shown, with the single tube partitioned into 3 segments. For each trial, 19 flies were initialized in segment 1, and a light source was shone on the distal end of segment 3. b) Graphs show the percentage of flies in each segment initially, after 15 min of darkness, and after 15 min of light. Statistical tests were carried out using ANOVA and Tukey HSD.

To assess phototaxis in wild-type and white-eyed *D. nebulosa*, we placed flies in a plastic tube segmented into 3, and quantified the average number of flies in each segment of the tube after dark and light conditions. All flies were initially placed in the proximal end of segment 1, and a light source was placed facing the distal end of segment 3 (Fig. 7a). We expected that flies kept in darkness would not show a tendency to travel to any specific part of the tube. Consequently, the distribution of the fly population would be random, and the number of flies at the distal end (segment 3) would not be significantly different between initial conditions and 15 min of darkness. However, flies with a positive phototactic response would be expected to travel toward the light source. Thus, the number of flies in segment 3 should be significantly greater after 15 min of light than compared with the amount after 15 min of darkness. The number of flies in segment 2 before (wt = 0, *w^−^* = 0) and after 15 min of darkness (wt = 3.3, *w^−^* = 1.0) showed no significant difference for both wild-type and white-eyed *D. nebulosa* (*P = *0.58, *P = *0.97). The same was true for number of flies in segment 3, before (wt = 0, *w^−^* = 0) and after 15 min of darkness (wt = 2.0, *w^−^* = 0.3). However, the number of flies in segment 3 was significantly greater (*P* = 0.02, *P* = 0.00) after 15 min of light (wt = 7.8, *w^−^* = 6.8), compared with the same segment after 15 min of dark conditions (Fig. 7b; Supplementary Table 6). This indicated that both wild-type and white-eyed *D. nebulosa* males respond to light.

## Discussion

In order to generate tools for genetic and genomic analyses in *D. nebulosa*, we assembled a long-read annotated *D. nebulosa* genome. This assembly is highly complete and contiguous and compares favorably to other genome assemblies in the *willistoni* clade. Using the genome of *D. willistoni* as a reference, we predicted that near-entire chromosomal arms can be reconstructed with ∼1–4 scaffolds from the *D. nebulosa* assembly. Scaffolds also span intergenic regions, facilitating the design of molecular experiments within the species. In particular, the assembly contiguity was outstanding relative to many available *Drosophila* genome assemblies. Comparison of N50 values across 26 *Drosophila* assemblies shows our assembly as the highest of the species sampled within its clade, with a contig size comparable to the current *D. melanogaster* assembly (Fig. 2). Assembly completeness was evaluated via BUSCO, with *D. nebulosa* showing scores in line with the other assembly references (Fig. 3; Supplementary Fig. 8).

Annotation BUSCO scores are comparable to that of the genome, indicating that our annotation likely captures the majority of protein coding genes in this species. The number of *D. nebulosa* genes (Table 1) annotated in the final version (iteration 3) is comparable to the number of *D. willistoni* protein coding genes in the current assembly annotation (GCF_000005925.1) (Clark *et al.* 2007; Zimin *et al.* 2008), which is expected for 2 species of the same subgroup. This comparison provides further confidence in our de novo assembly.

Phylogenomic trees inferred using supermatrix (Fig. 2) and supertree approaches (Supplementary Fig. 2) recovered identical tree topologies and placed *D. nebulosa* as sister to a clade containing *D. willistoni*, *D. paulistorum*, *D. tropicalis* and *D. insularis*, and within the monophyletic *willistoni* group (van der Linde and Houle 2008). We recover a nonsister relationship between *D. nebulosa* and *D. sucinea*. This finding supports previous work suggesting paraphyly of the *bocainensis* subgroup (Gleason *et al.* 1998; Tarrio *et al.* 2000; Zanini *et al.* 2018).

In the several species of the *willistoni* group, the dot chromosome does not exist alone and is instead fused to chromosome 3 (fusion of Muller elements E + F). Previous studies have used fluorescence in situ hybridization to demonstrate that the *ey*, *ci*, and *Ankyrin* (*Ank)* genes, which are present on chromosome 4 in *D. melanogaster*, are part of chromosome 3 in a number of species in the *willistoni* and *bocainensis* subgroups, *D. willistoni* and *D. nebulosa* included (Papaceit and Juan 1998; Pita *et al.* 2014). Hence, *D. nebulosa* has 3 chromosomes: X, 2, and 3; with the X and 2 consisting of a left and right arms (Pavan 1946; Valente *et al.* 1996; Schaeffer *et al.* 2008).

As an attempt to correlate some of the larger scaffolds to their potential chromosome, we searched for syntenic regions between *D. nebulosa* and *D. willistoni* assemblies (Fig. 5). Altogether, this evidence suggests that the *D. nebulosa* assembly succeeded in generating scaffolds that are congruent with known *D. willistoni* chromosomal arms 2L (dneb_sca_0), 2R (dneb_sca_1), 3 (dneb_sca_3, 6, and 10), XL (dneb_sca_2, 5, and 9), and XR (dneb_sca_2, 4, 7, and 8). These data show that the largest 11 scaffolds from the assembly account for all 3 *D. nebulosa* chromosomes (5 chromosome arms). Additionally, the *ey*, *ci*, and *Ank* genes are all on sca_3 in the *D. nebulosa* assembly. This reflects the fusion of the dot chromosome and chromosome 3 and is in agreement with experimental results of previous studies (Papaceit and Juan 1998; Pita *et al.* 2014). Scaffolds constituting the X chromosome appear to be less contiguous, most likely due to homologous, but divergent X and Y gametologs from the mix of male and female *D. nebulosa* used for sequencing. As of note, the assignment of *D. willistoni* chromosome 2 arms have been debated (Rohde *et al.* 1995; Schaeffer *et al.* 2008; Garcia *et al.* 2015), but for the purposes of this discussion, the study by Garcia *et al.* (2015) was mainly referenced. Accordingly, it should be noted that assigning scaffolds to chromosomes is based on synteny with *D. willistoni*.

The genome of *D. nebulosa* has long been established to contain many instances of chromosomal rearrangements (Pavan 1946; Valente *et al.* 1996; Papaceit and Juan 1998; Pita *et al.* 2014). In addition, genetic recombination in the X chromosome is more frequent than in autosomes (Rius *et al.* 2016). This may account for the syntenic variation we see in reference to the *D. willistoni* assemblies, such as the rearrangement of specific regions between chromosome arms XL and XR, or XR and 2R (Fig. 5). Although this variation could be due to contig mis-joining in the reference assemblies, interspecies chromosomal variation has been previously characterized in *D. willistoni* (Rohde and Valente 2012). Overall, the latter possibility is favored since both reference *D. willistoni* assemblies (*wil_caf1* and *wil_17*) are from different isolates (Supplementary Table 8).

As a final metric of assembly contiguity and completeness, we set to assess how well scaffolds can recapitulate *D. nebulosa* chromosomal arms. One measure of this is whether the assembly scaffolds include centromeric regions at the end of the chromosome. In drosophilids, transposable elements have been shown to be distributed more densely in centro- and telomeric regions, as well as other regions of low recombination rate (Thomas *et al.* 2015; Rius *et al.* 2016). Indeed, we see high density regions of transposable elements at the ends of 5 *D*. *nebulosa* scaffolds that are predicted to each comprise different chromosome arms (Fig. 5). Furthermore, MAKER repeat annotation did not show a high density of HeT-A, TART, or TAHRE retrotransposable elements, which are known to constitute *Drosophilid* telomeres. Altogether, it provides evidence that scaffolds include sequence up to centromeric regions.

Using CRISPR/Cas9 genome editing has a great potential to develop new and powerful model organisms to address evolutionary processes related to cell signaling, tissue patterning, morphogenesis, and behavior. In addition, it would bypass years of mutation-induced screens, as was done for many alleles found in *D. melanogaster*. In *D. melanogaster*, white-eyed flies are commonly used for transgenic experiments. To our knowledge, this is the first time CRISPR/Cas9 was successfully utilized in *D. nebulosa* by targeting *w* in the genome (Fig. 6b). At the same time, characterization of the disrupted locus revealed that although flies injected with both guides were positive for disrupted *w*, only one of the 2 PAM targets was cut in every case (Fig. 6c). Interestingly, using both guide RNAs generated 9 different deletions in the gene (Fig. 6, g and h). This strategy can potentially serve as a tool to generate different alleles and let selection act on a viability scale. While these were different types of deletions, none could produce a viable white-eyed female fly.

Short indels proximal to the targeted PAM sites are indicative of nonhomologous end joining, as opposed to homology-directed repair (Gratz *et al.* 2013). This is further supported by the fact that the *nos*-*Cas*9 cassette, designed to integrate within the *w* gene, was not present in any of the tested lines (Fig. 6, d–f). The role of the donor vector is to repair double strand breaks created by Cas9. One possible reason for this is that the homology-directed repair pathway is known to be less efficient than the nonhomologous end joining (Roy *et al.* 2018). Therefore, breaks in the genome may have been ligated together before the donor vector was able to repair them. Previous studies have used various methods to increase CRISPR efficiency, such as *piggyBac*-mediated integration of the *nos*-*Cas9* locus (Gratz *et al.* 2014; Nishizawa-Yokoi and Toki 2021), inhibition of nonhomologous end joining pathway (Maruyama *et al.* 2015), and timed embryo injection with *in vivo* sgRNA efficiency (Kotwica-Rolinska *et al.* 2019), providing several options for future improving homology-directed repair efficiency in *D. nebulosa*. Altogether, we show that CRISPR/Cas9 can work in *D. nebulosa*. However, additional considerations will need to be implemented prior to becoming an efficient genetic model system.

Several *Drosophila* species are available as viable white-eyed stocks and used in transgenic experiments (Holtzman *et al.* 2010; Stern *et al.* 2017). However, most of these species court via acoustic, chemical, and tactical modalities and are not solely dependent on vision. In contrast, mating in several species has been found to require vision (Jezovit *et al.* 2017; Keesey *et al.* 2019). For example, *D. nebulosa* males initiate courtship by uppercutting the female with their legs, standing perpendicularly and angling their posterior toward her, and silently fanning an extruded anal droplet in her direction via flicking motions with one wing. In the absence of light, *D. nebulosa* males were unable to orient themselves toward the female, resulting copulation failure (Gleason *et al.* 2012). In species such as *D. nebulosa* and others like it, we expected that impairing visual acuity and optical insulation through disruption of *w* (Ferreiro *et al.* 2017) would jeopardize courtship rituals, and thus copulation. While commonly used, white-eyed *D. melanogaster* demonstrated reduced courtship. The phenotype was shown to be alleviated in these flies with the introduction of the mini-*w* gene (Xiao *et al.* 2017). It is possible that copulation was still successful in white-eyed *D. melanogaster*, since males court using wing-vibrations to produce a species-specific “song” (Spieth 1952). Conversely, disruption of *w* in *D. suzukii*, whose courtship rituals are more similar to *D. nebulosa*, resulted in no attempts at courtship or copulation (Yan *et al.* 2020).

In our study, pairing white-eyed males with virgin wild-type *D. nebulosa* females produced eggs, but never any larva. Consequently, white-eyed females were never observed in any of the lines. We predicted that male and female crosses failed to copulate, thus leading to unfertilized eggs. To support this, we compared single crosses of a white-eyed and wild-type *D. nebulosa* male paired with a virgin female. A clear difference in courtship display was prevalent between the crosses of wild-type *D. nebulosa*, where males frequently attempted courting females and exploring the vial. This observation is in contrast to the white-eyed males, which did not attempt any courting, even when approached by females. In fact, these males rarely move at all in the vials. In light of these observations, and the established mating behavior of *D. nebulosa*, it is possible that white-eyed males are unable to visually locate the female.

One way to assess the extent of lowered visual acuity is to examine the effects of the disrupted *w* gene on phototactic response in *D. nebulosa*. The expectation would be that wild-type *D. nebulosa* capable of perceiving light would be attracted to it and cluster near the source. Conversely, *D. nebulosa* that are completely blind would be expected to be ignorant to the light source, and thus disperse randomly. Furthermore, phototactic success in white-eyed flies due to perception via ocelli is also unlikely, since *w* is required for pigmentation in the eyes, as well as the ocelli (Levis *et al.* 1985; Caldwell *et al.* 2007). Our findings showed that similarly to wild-type *D. nebulosa*, white-eyed flies were attracted to light and traveled toward the source (Fig. 7b). These data indicate that white-eyed *D. nebulosa* can perceive light but perhaps lack the visual acuity to locate potential mates. It should also be noted that impaired vision may not fully account for the failure to court in white-eyed *D. nebulosa*. White protein also is responsible for transporting precursors of neurotransmitters across cell membranes, such as serotonin and dopamine. As such, studies have suggested that abnormal levels of neurotransmitters underlie mating irregularities, such as decreased copulation rate (Xiao *et al.* 2017) and enhanced male–male courtship (Krstic *et al.* 2013) in *D. melanogaster* null for and ectopically overexpressing *w*, respectively.

The *D. nebulosa* species provides a compelling model system to investigate a variety of biological phenomena, such as evolution of cell signaling, patterning, morphology (Niepielko *et al.* 2012), chromosomal arrangements (Valente *et al.* 1996), mating behavior (Gleason *et al.* 2012), and even radiation resistance (Kratz 1975). Here we suggest that, given the fundamental evolutionary differences in *D. nebulosa’*s courtship, this species is an attractive organism to develop genetic tools to study the visual requirements underlying mating success. Unlike the challenges to rear *D. willistoni* (Holtzman *et al.* 2010), *D. nebulosa* is simple to rear in the same conditions as *D. melanogaster*. However, the disruption of the visual system, which is required for mating, should be avoided, and other phenotypic markers should be considered that are not involved with vision. A potential solution is to choose a selectable marker which is not involved in vision and behavior. One such possibility is the wing marker, *crossveinless* (*cv*) {CG12410, FBgn0000394}. This gene is known to be on the X chromosome in *D. melanogaster*. In our *D. nebulosa* assembly, *cv* has CDS length of 633 bp, and is found on *dneb_sca_5* (predicted to belong to the chromosomal arm XL). The gene’s small size would make it easier to clone into vectors for phenotypic rescue (Shimmi *et al.* 2005), and like *white*, sex-linkage would allow us to screen for males of the F1 generation.

## Supplementary Material

jkac231_Supplementary_Figure_S1

jkac231_Supplementary_Figure_S2

jkac231_Supplementary_Tables_S1_S9_S11_S14

jkac231_Supplementary_Tables_S10

## Data Availability

This Whole Genome Shotgun project has been deposited at DDBJ/ENA/GenBank under the accession JANFPS000000000. The version described in this paper is version JANFPS010000000. The raw data can be found by the SRA accession numbers and SRR20301698 and SRR20301698 for PacBio and Illumina reads, respectively. The genome annotation has been deposited at Harvard Dataverse under the doi:10.7910/DVN/JOVNWY. Supplemental material is available at G3 online.
