# Supplementary material for: Nebulous without white: annotated long-read genome assembly and CRISPR/Cas9 genome engineering in Drosophila nebulosa
Source: G3 (Bethesda). 2022 Sep 5;12(11):jkac231. doi: 10.1093/g3journal/jkac231 (PMC9635631; doi:10.1093/g3journal/jkac231)
Supplement: jkac231_Supplementary_Figure_S1 [file jkac231_supplementary_figure_s1.pdf]

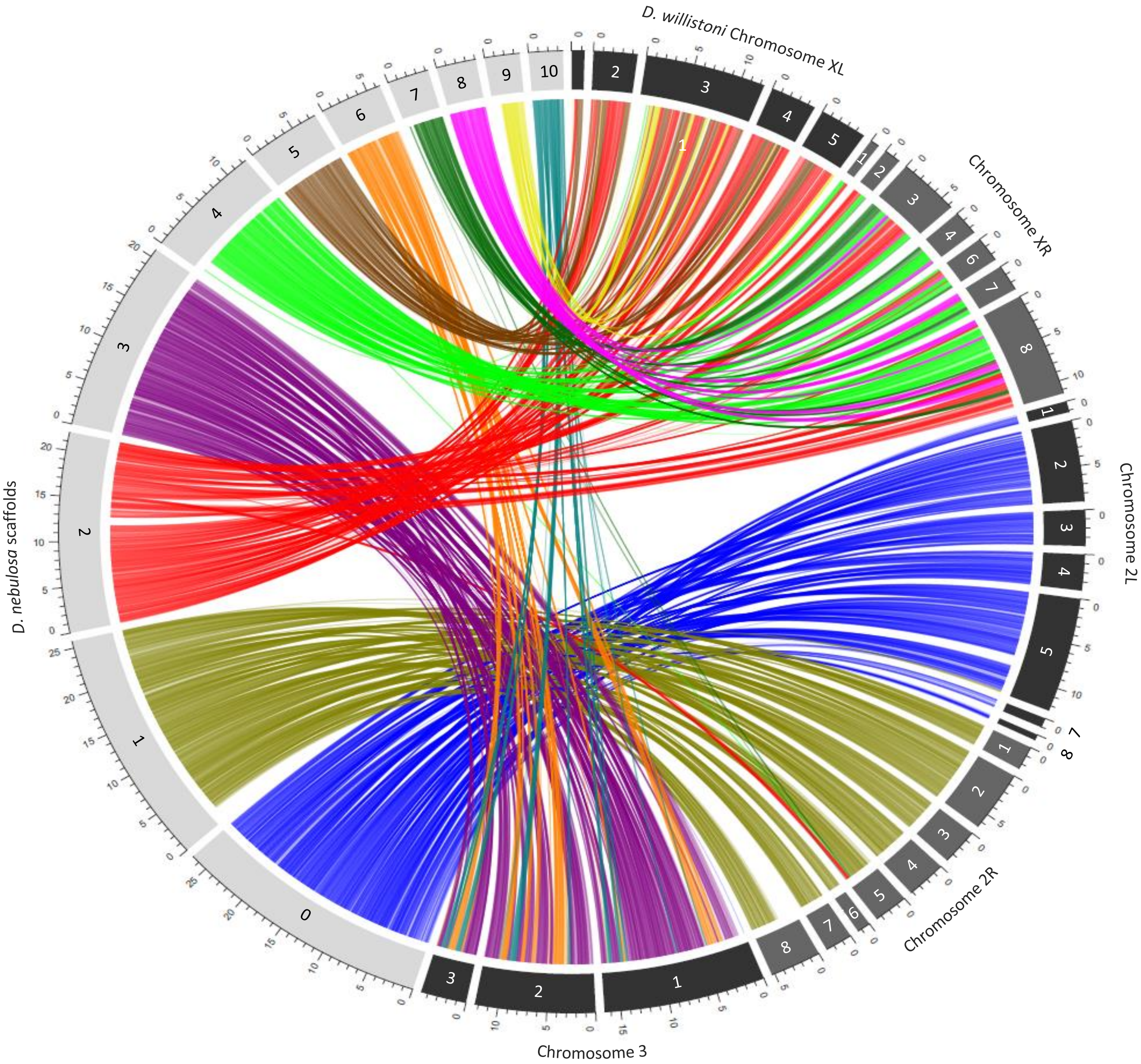

Figure S1. Comparison of chromosome synteny between *D. willistoni* and *D. nebulosa* scaffolds. Annotated *D. willistoni* caf1 scaffolds are grouped by chromosome (indicated by the black and dark gray) and compared to the eleven largest *D. nebulosa* scaffolds (light gray). Syntenic regions between the two assemblies are represented by the then curved lines. Line color represents synteny with an associated *D. nebulosa* scaffold. Scaffold names from the top are listed in clockwise fashion: *D. willistoni* 17 ChrXL_1 - 5, ChrXR_1 - 6, 7 - 8, Chr2L_1 - 5, 7 - 8, Chr2R_1 - 8, Chr3_1 - 3, and dneb_sca_0 - 10.
