# Supplementary material for: Nebulous without white: annotated long-read genome assembly and CRISPR/Cas9 genome engineering in Drosophila nebulosa
Source: G3 (Bethesda). 2022 Sep 5;12(11):jkac231. doi: 10.1093/g3journal/jkac231 (PMC9635631; doi:10.1093/g3journal/jkac231)
Supplement: jkac231_Supplementary_Figure_S2 [file jkac231_supplementary_figure_s2.pdf]

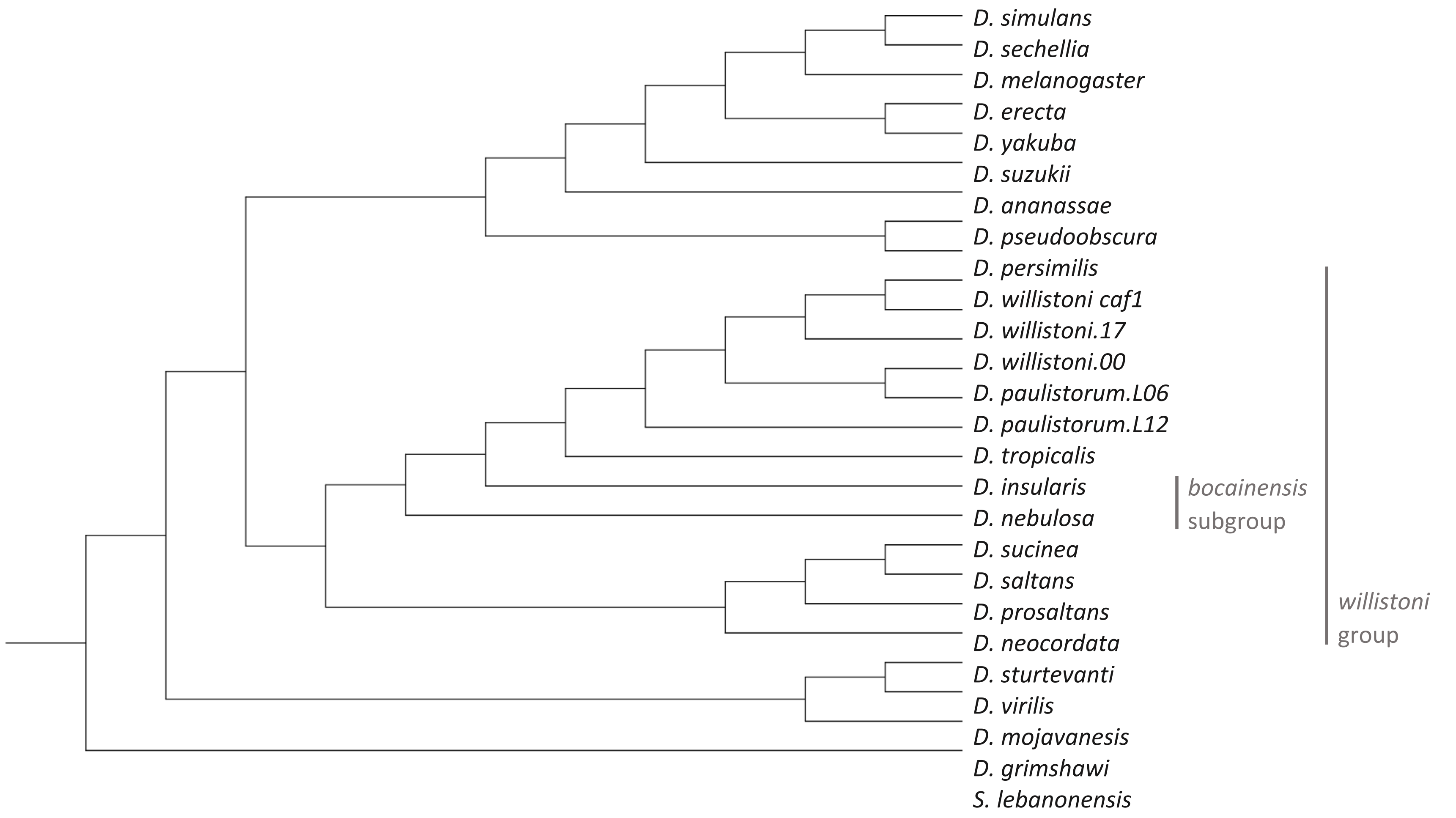

Figure S2. Phylogenomic tree of the *Drosophila* genus based on supertree methods. Topology was inferred via concatenation of 3285 Universal Single Copy Orthologs present in all lineages, and rooted with *Scaptodrosophila lebanonensis* . Bootstrap values were 100% for all branches.
